# The complete mitogenome of the granular torrent frog, *Amolops granulosus* (Anura: Ranidae)

**DOI:** 10.1080/23802359.2019.1643800

**Published:** 2019-07-19

**Authors:** An Huang, Hongdi Luo, Sangdawa Luo, Haijun Li, Qingyong Ni, Yongfang Yao, Huailiang Xu, Bo Zeng, Ying Li, Zhimin Wei, Mingwang Zhang

**Affiliations:** a Farm Animal Genetic Resources Exploration and Innovation Key Laboratory of Sichuan Province, Sichuan Agricultural University, Chengdu, China;; b College of Animal Sciences and Technology, Sichuan Agricultural University, Chengdu, China;; c Forestry and Grassland Administration of Qushui County, Lhasa, China;; d College of Life Science, Sichuan Agricultural University, Ya’an, China;; e Institute of Millet Crops, Hebei Academy of Agriculture and Forestry Sciences, Shijiazhuang, China

**Keywords:** Mitochondrial genome, gene arrangement, *Amolops granulosus*

## Abstract

We obtained the complete mitochondrial genome of *Amolops granulosus*, which was 17,785 bp in length and it contained the 37 typical mitochondrial genes: 2 ribosomal RNAs, 22 transfer RNAs (tRNAs), 13 protein-coding genes (PCGs), and 1 control region (CR). The hotspot of gene arrangement was ranged as ‘W-gap-O_L_-gap-A-N-gap-C-Y’ which consisted with most published *Amolops* mitogenomes. Our phylogenetic results suggested the gene arrangement of ‘WANCY’ region can facilitate to distinguish the *Amolops* species as an efficient genetic marker.

The genus *Amolops* is distributed across Nepal, northern India, western and southern China to Malaya and contains 58 species (Frost 2019). Recently, some mitochondrial genomes of genus *Amolops* have been reported that the tRNA gene cluster, ‘WANCY’, as a hotspot of mtDNA rearrangement, and it has provided evidence of rearrangements between species within the genus (Huang et al. 2014; Shan et al. 2016; Xue et al. 2016; Zhang et al. 2016). But the phylogenetic relationship of these species still remains controversial. In order to evaluate its phylogenetic position in the genus *Amolops*, we sequenced the complete mitochondrial genome of *Amolops granulosus*, a torrent frog which is an endemic species to China.

The sample was collected from Wawushan Mountain (29°38′38.98″ N, 102°55′48.06″ E, Sichuan, China) and placed in the College of Animal Sciences and Technology, Sichuan Agricultural University, China (voucher 20130258). Total genomic DNA was extracted from the muscle tissue using Ezup type animal genomic DNA extraction kit (Sangon, Shanghai) according to the manufacturer’s protocols. The DNA samples were sent to Personal Biotechnology Company (Shanghai, China) for library preparation and Illumina sequencing using a 2 × 251 paired-end strategy. The assembled sequence was annotated using MITOS web server (Bernt et al. 2013) and manually identified by comparing with related databases from GenBank.

In this study, the complete mitogenome of *A. granulosus* was 17,785 bp in length (GenBank Number: MH922934) and it contained the 37 typical genes: 2 ribosomal RNAs, 22 transfer RNAs (tRNAs), 13 protein-coding genes (PCGs), and a control region (CR). The length of 12S rRNA was 933 bp and of 16S rRNA was 1587 bp, while 22 tRNAs ranged from 65 to 72 bp, which were similar to other *Amolops* species. Among the 13 PCGs, most genes used ATG as an initiate codon, except for *ND2* and *COI*, which started with ATT and ATA, respectively. For the stop codon, *COI* and *ND5* ended with AGG, *ND6* ended with AGA, *ND2* and *ATP8* ended with TAG, *ND4L* and *Cytb* stopped with TAA, while the remaining PCGs shared the incomplete terminal codon (TA or T). The hotspot of gene arrangement was ranged as ‘W-gap-O_L_-gap-A-N-gap-C-Y’ in the mitochondrial genome of *A. granulosus*, which consisted most published *Amolops* mitogenomes, with the exception of *Amolops ricketti* and *Amolops wuyiensis.*

Based on 13 PCGs from 26 Anura species, we depicted the molecular phylogeny in Figure 1. The best-fit nucleotide substitution models were determined using Partitionfinder (Lanfear et al. 2017). Bayesian analyses were conducted using MrBayes 3.2.1 with the Marko chain Monte Carlo (MCMC) for 10,000,000 generations and 1000 sampled generations (Ronquist et al. 2012). The phylogenetic tree showed that the seven *Amolops* species were divided into two clades, monophyly of (*Amolops loloensis + Amolops tuberodepressus* + *Amolops mantzorum + Amolops chunganensis + A. granulosus*) and (*Amolops ricketti* + *Amolops wuyiensis*) was strongly supported. Our phylogenetic results were not only congruent with previous studies, but also suggested the gene arrangement of ‘WANCY’ region can facilitate to distinguish the *Amolops* species as an efficient genetic marker (Xue et al. 2016; Yuan et al. 2016).

**Figure 1.**
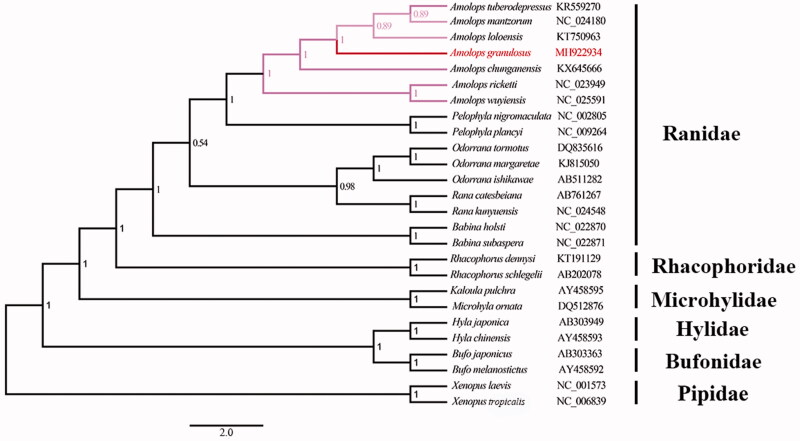
A phylogenetic tree based on the concatenated nucleotide sequences of 13 PCGs from 26 Anura species constructed with Bayesian inference (BI). *Xenopus laevis* and *X. tropicalis* were used as outgroups. Bayesian posterior probabilities are shown near the nodes.
